# Exopolysaccharide-mediated surface penetration as new virulence trait in *Enterococcus faecalis*

**DOI:** 10.1080/19420889.2019.1657373

**Published:** 2019-08-26

**Authors:** Yusibeska Ramos, Diana K. Morales

**Affiliations:** Department of Obstetrics and Gynecology, Weill Cornell Medicine, New York, NY, USA

**Keywords:** Enterococcus faecalis, polyGlcNAc-containing exopolysaccharides, semisolid surfaces, epithelial cell monolayers

## Abstract

*Enterococcus faecalis* is a commensal bacterium that normally inhabits the gastrointestinal tract of humans. This non-motile microorganism can also cause lethal infections in other organs by penetrating and breaching the intestinal barrier. However, the precise molecular mechanisms enabling *E. faecalis* movement and translocation across epithelial barriers remain incompletely characterized. We recently reported that *E. faecalis* utilizes the RpiA-GlnA-EpaX metabolic axis to generate β-1,6-linked poly-*N*-acetylglucosamine (polyGlcNAc)-containing exopolymers that are necessary for its optimal migration into semisolid surfaces and efficient translocation through human epithelial cell monolayers. These findings provide new evidence indicating that non-motile bacterial pathogens can exploit carbohydrate metabolism to penetrate surfaces. Hence, targeting this process might represent a new strategy to more effectively control systemic infections by *E. faecalis*.

*Enterococcus faecalis* is a non-motile bacterium capable of colonizing the gastrointestinal tract (GI) of healthy humans. Importantly, this microbe has the capacity to switch from commensal to pathogen, which results in life-threatening infections [1]. *E. faecalis* ranks as the fourth most important cause of hospital-acquired infections and the third leading cause of bacteremia, where its intrinsic or acquired antibiotic resistance contributes to its pathogenicity [2]. Recent evidence suggests that *E. faecalis* has a remarkable ability to translocate from the GI to reach the bloodstream and colonize distant anatomical locations [3–6]. For instance, in patients treated with antibiotics during allogeneic hematopoietic stem cell transplantation, colonization of the intestinal tract by antibiotic-resistant enterococci has been observed prior to the emergence of bloodstream infections [4]. Moreover, the use of broad-spectrum antimicrobials has been suggested to be a predisposing factor that promotes the translocation of vancomycin-resistant enterococci [5]. While the mechanisms of enterococcal migration remain largely unexplored, recent evidence indicates that this process is mediated by the formation of bacterial aggregates through cell-surface appendages, adhesins, and other unknown factors [4–10]. A deeper understanding of the molecular processes that enable *E. faecalis* movement and translocation across epithelial barriers is required to more effectively control systemic infections by this opportunistic pathogen.

Our group recently uncovered a new mechanism used by *E. faecalis* to penetrate semisolid surfaces and translocate through human epithelial cell monolayers, where synthesis and secretion of enterococcal extracellular polysaccharides is requiered [8]. When grown on semisolid agar, *E. faecalis* cells penetrated and invaded into the medium, thereby creating a “*colony-print*” (penetrating cells;). Strikingly, some cells remained on the surface of the media (non-penetrating cells) and developed external and regular colonies (Figure 1 (a)). Since we identified this semisolid penetration trait in several clinical isolates, as well as in the commensal-like strain *E. faecalis* OG1RF, we used this assay to dissect the molecular mechanisms utilized by this bacterium to move into surfaces. Interestingly, a similar penetration ability was previously described in the fungus *Candida albicans* [11], suggesting that this process could be a conserved strategy used by non-motile opportunistic pathogens.

**Figure 1.**
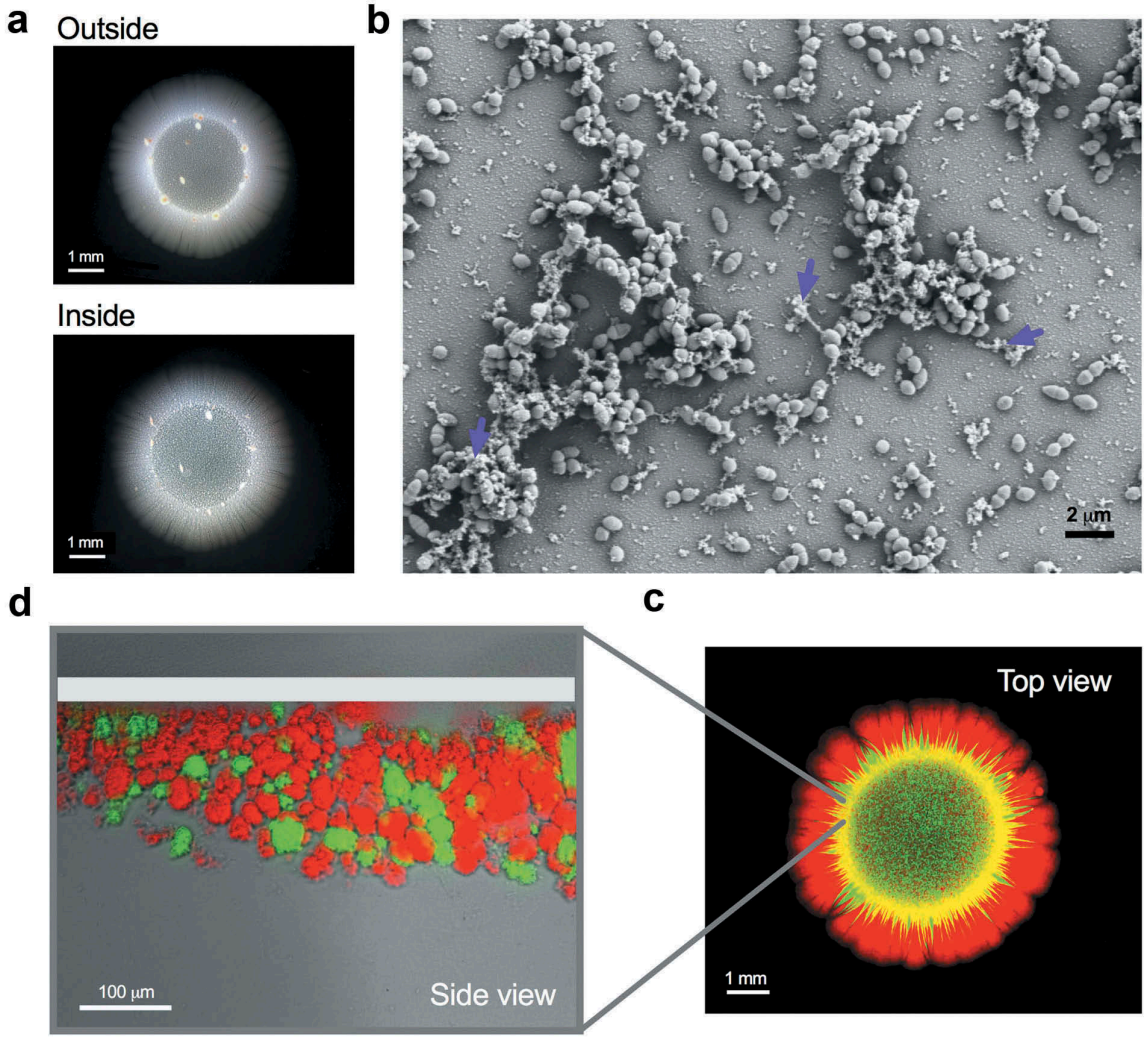
Enterococcal surface penetration. (a–c). The capacity of *E. faecalis* MMH594 to penetrate was evidenced as a colony-print inside the agar after removing the non-penetrating cells (outside) through several washes with water. (a) Images of a 6-day-old colony (outside) and its penetrating cells (inside) grown on semisolid medium at 37°C. (b) Scanning electron microscopy of aggregated and matrix-covered (*purple arrow head*) enterococcal cells. (c) Top view of a colony and its invading community (side view) demonstrating the spatial localization of discrete *E. faecalis* aggregated communities differentially labeled with either red (m-Cherry) or green (GFP) fluorescent proteins. The top white line indicates the beginning of the agar. Scale bars: 1 mm (a and c); 2 μm (b) and 100 μm (d). Methodological details are further described in reference [8].

In our efforts to unravel the *E. faecalis* penetration process, we sought to characterize the differences between penetrating and non-penetrating cells. We found that both populations were formed by diplococci and remained viable after six days of growth. However, penetrating cells assembled complex multicellular aggregates that were covered with and connected by an abundantly produced extracellular matrix (Figure 1(b)). Our results demonstrated that this matrix was partly composed by β-1,6-linked poly-*N*-acetylglucosamine (polyGlcNAc)-containing polymers. Further, we found that the absence of these polysaccharides rendered *E. faecalis* incapable of efficiently penetrating the semisolid surface. We determined that *E. faecalis* aggregates were formed by metabolically active cells [8] and exhibited an organized spatial distribution. When two *E. faecalis* strains constitutively expressing m-Cherry or GFP reporters were co-cultured, spatially-differentiated red or green regions, respectively, were observed within the penetrating colony print (Figure 1(c)), suggesting that an unknown process might dictate the cellular localization within the aggregates.

Mechanistically, our study revealed that the RpiA-GlnA-EpaX metabolic axis enabled the synthesis of polyGlcNAc-containing polysaccharides that were necessary for *E. faecalis* surface penetration. GlnA (glutamine synthetase A) and RpiA (ribose-5-phosphate isomerase A) participate in key metabolic pathways implicated in this process. GlnA catalyzes the condensation of glutamate and ammonia to generate glutamine [12], while RpiA is involved in the reversible conversion of ribose-5-phosphate to ribulose-5-phosphate, a central enzymatic reaction in the pentose phosphate pathway [13]. EpaX, on the other hand, is a putative glycosyltransferase that is necessary for the transfer of glycosides onto *E. faecalis* polysaccharides [14]. Our results linked the metabolic functions of GlnA and RpiA with the hexosamine biosynthetic pathway. We proposed that the glutamine produced by GlnA, together with fructose-6-phosphate generated from metabolites of the pentose phosphate pathway, are used as building blocks to form the uridine diphosphate *N*-acetylglucosamine (UDP-GlcNAc) that EpaX needs to produce polyGlcNAc-containing extracellular polysaccharides. Since our work showed that exogenous addition of polyGlcNAc-containing polysaccharides restored the ability of a defective strain to penetrate semisolid surfaces, we envisioned a possible conserved mechanism used by *E. faecalis* to migrate through surfaces such as epithelial barriers to reach other body anatomical sites. Indeed, we demonstrated that the RpiA-GlnA-EpaX metabolic axis was also necessary for movement through human intestinal barriers, and this process involved the formation of bacterial aggregates. Thus, strains devoid of either GlnA, RpiA or EpaX had impaired ability to translocate through epithelial cell monolayers [8]. Altogether these results revealed a new role for polyGlcNAc-containing extracellular polysaccharides as key mediators of *E. faecalis* migration traits.

Previously work has demonstrated that polysaccharides are key elements of bacterial pathogenicity [14–19]. In *E. faecalis*, the enterococcal polysaccharide antigen (EPA), has been shown to be essential for bacterial adhesion to intestinal mucus, translocation through epithelial cells, biofilm formation, resistance to antibiotics and to phagocytosis, colonization, and virulence in several animal models [14,15,20–23]. Moreover, genes encoding proteins necessary for EPA synthesis or decoration have been demonstrated to be crucial for *E. faecalis* persistence and adaptation by helping this bacterium tolerate intestinal environmental stresses [24]. Chatterjee and collaborators recently demonstrated that mutations in genes involved in decorating EPA reduced *E. faecalis* resistance to antibiotics, but conferred elevated resistance to phage infections in both cultures and in an intestinal colonization mouse model [25]. Our work demonstrated that the absence of either a polyGlcNAc decoration of EPA or a new polyGlcNAc-containing polysaccharide impaired the ability of *E. faecalis* to move into surfaces, suggesting a new role for these exopolysaccharides [8].

An intriguing question that remains to be answered is how *E. faecalis* uses these polyGlcNAc-containing exopolymers as a migration strategy. In *Bacillus subtilis*, for instance, it has been shown that production of a polysaccharide-containing extracellular matrix drives spreading of the cells by generating osmotic pressure gradients in the extracellular space within biofilms [26]. This phenomenon has been also described in *Proteus mirabilis*, which secretes polysaccharides to create a fluidic environment that promotes movement on low moisture surfaces. In this bacterium, the absence of exopolysaccharides changed the osmotic ratio and reduced the migration velocity [27]. In *E. faecalis*, polyGlcNAc-containing polymers might help cells aggregate to facilitate their movement. Indeed, it has been shown that polyGlcNAc matrices promote cell-cell contact by enabling intercellular adhesion by electrostatic interactions between cells surfaces [28]. Our results support the idea that polyGlcNAc-containing polymers might act as a lubricant to reduce surface friction and help *E. faecalis* spread by establishing a fluidic environment through which bacteria can slide and penetrate surfaces.

Synthesis and secretion of polyGlcNAc-containing polymers by *E. faecalis* may represent an evolutionary advantage that could allow this non-motile bacterium to reach nutrients or escape stressful environments. In fact, bacteria forming polysaccharide-covered multicellular aggregates exhibit fitness benefits and cooperative behavior where cell-cell aggregation increases both the “per-capita” availability of resources and the per-cell growth rate [29]. Thus, we proposed that polyGlcNAc-containing exopolymers help *E. faecalis* cells not only to aggregate, but also to behave as a coordinated community where few members become “*explorers*” by moving into and invading new and unexplored environments.

In summary, our recent work demonstrated that *E. faecalis* utilizes the RpiA-GlnA-EpaX axis to generate polyGlcNAc-containing exopolysaccharides necessary for optimal migration into semisolid surfaces and for efficient translocation through human epithelial cell monolayers. These new mechanisms open the possibility that exopolymer-mediated migration could operate as a common physiological trait that might help non-motile cocci to penetrate and explore new environments. Hence, our recent findings revealed a previously unappreciated mechanism of virulence in *E. faecalis* that might be targeted to more effectively control systemic infections by this pathogen.
